# Supplementary material for: Dissipation Behavior and Dietary Risk Assessment of Thiamethoxam, Pyraclostrobin, and Their Metabolites in Home-Style Pickled Cowpea
Source: Foods. 2023 Sep 6;12(18):3337. doi: 10.3390/foods12183337 (PMC10527991; doi:10.3390/foods12183337)
Supplement: Supplementary file 1 [file foods-12-03337-s001.zip › foods-2567763-supplementary.pdf]

Supplementary Materials:

Table S1. Mass spectrometry parameters of THI, CLO, PYR, BF 500-3.

Compound Name	Retention time (min)	Parent (m/z)	Daughter (m/z)	Cone (v)	Collision (v)
THI	1.11	292.0000	132.0000	25	20
		292.0000	211.2000[※]	25	15
CLO	1.13	250.1000	168.8700[※]	24	12
		250.1000	131.8600	24	14
PYR	1.46	388.0500	163.2000[※]	18	28
		388.0500	194.2500	18	12
BF 500-3	1.47	357.9681	132.0170[※]	18	36
		357.9681	164.0662	18	14
